# Dispersion and new shelters offered by ants: myrmecophoresy of tardigrades

**DOI:** 10.1186/s12983-025-00581-3

**Published:** 2025-10-06

**Authors:** Daniele Giannetti, Ilaria Giovannini, Edoardo Massa, Enrico Schifani, Lorena Rebecchi, Roberto Guidetti, Donato A. Grasso

**Affiliations:** 1 Department of Chemistry, Life Sciences and Environmental Sustainability, University of Parma, Parco Area delle Scienze 11/A, 43124 Parma, Italy; 2 Department of Life Sciences, University of Modena and Reggio Emilia, 41125 Modena, Italy; 3 National Biodiversity Future Center (NBFC), 90133 Palermo, Italy

**Keywords:** Insects, Micrometazoans, Phoresy, Galls, Mosses, Behavior, Symbiosis

## Abstract

The present study investigates the potential role of ants as dispersal hosts for tardigrades and for the first time provides evidence of ant-mediated tardigrade phoresy. Tardigrades are microscopic cosmopolitan animals which have limited autonomous dispersal abilities but can withstand extreme conditions in a desiccated state. Being dominant terrestrial organisms, ants interact with many components of ecosystems, yet their role in dispersing meiofaunal organisms is unknown. In a field survey, four arboreal ant species were first analyzed to test the presence of tardigrades in their nests (*i.e.* tree galls), and on their bodies. In another experiment, galls were maintained isolated, then exposed to ant colonization to evaluate any transport of tardigrades by ants. Finally, the behavior of the ant *Colobopsis truncata* was tested by crafting an experimental apparatus to verify the actual phoresy of tardigrades. The field survey and gall colonization experiments show an association of tardigrades, especially with *C. truncata*. Gall colonization and laboratory experiments reveal that the ants transport tardigrades and other meiofaunal organisms, such as nematodes and rotifers. This phoresy can be direct (transporting animals) or indirect (transporting substrates with animals), over significant distances, thereby suggesting an unknown ecological interaction. Thanks to the widespread presence and abundance of ant species, this myrmecophoretic dispersion could play a crucial role in the spreading of meiofaunal organisms in terrestrial environments. These findings may represent just the ‘tip of the iceberg’ of an unexplored passive dispersal modality for terrestrial meiofauna micrometazoans, expanding our knowledge of phoretic relationships.

## Introduction

Phoresy is a special type of temporary symbiotic relationship in which the phoront, an organism with limited active dispersal abilities, uses a highly mobile dispersal host for transportation to new habitats or resources. In the context of current climate changes, such as habitat fragmentation or climate breakdown, phoresy is a dispersal strategy that can play a critical role for species persistence and resilience. Species dispersing phoretically have been observed in at least 13 animal phyla, 25 classes, and 60 orders [1]. Among these taxa, many species including some invertebrates have evolved behavioral strategies to interact with ants (Insecta; Hymenoptera; Formicidae). As typical examples, spider *Attacobius attarum* (Roewer, 1935) (Araneae; Corrinidae) and cockroaches *Attaphila fungicola* Wheeler, 1900 (Blattodea; Blaberidae) evolved a ‘hitchhiking’ dispersal strategy using winged ant queens [2, 3].

Ants are a widely distributed group of insects comprising over 14,212 species [4], and play a key role in terrestrial ecosystems [5, 6]. Their extensive diversification arises from different evolutionary strategies and often complex lifestyles, which allow the different ant species to interact, compete, and coexist in different ways within the same habitat [5, 7–11].

**Fig. 1:**
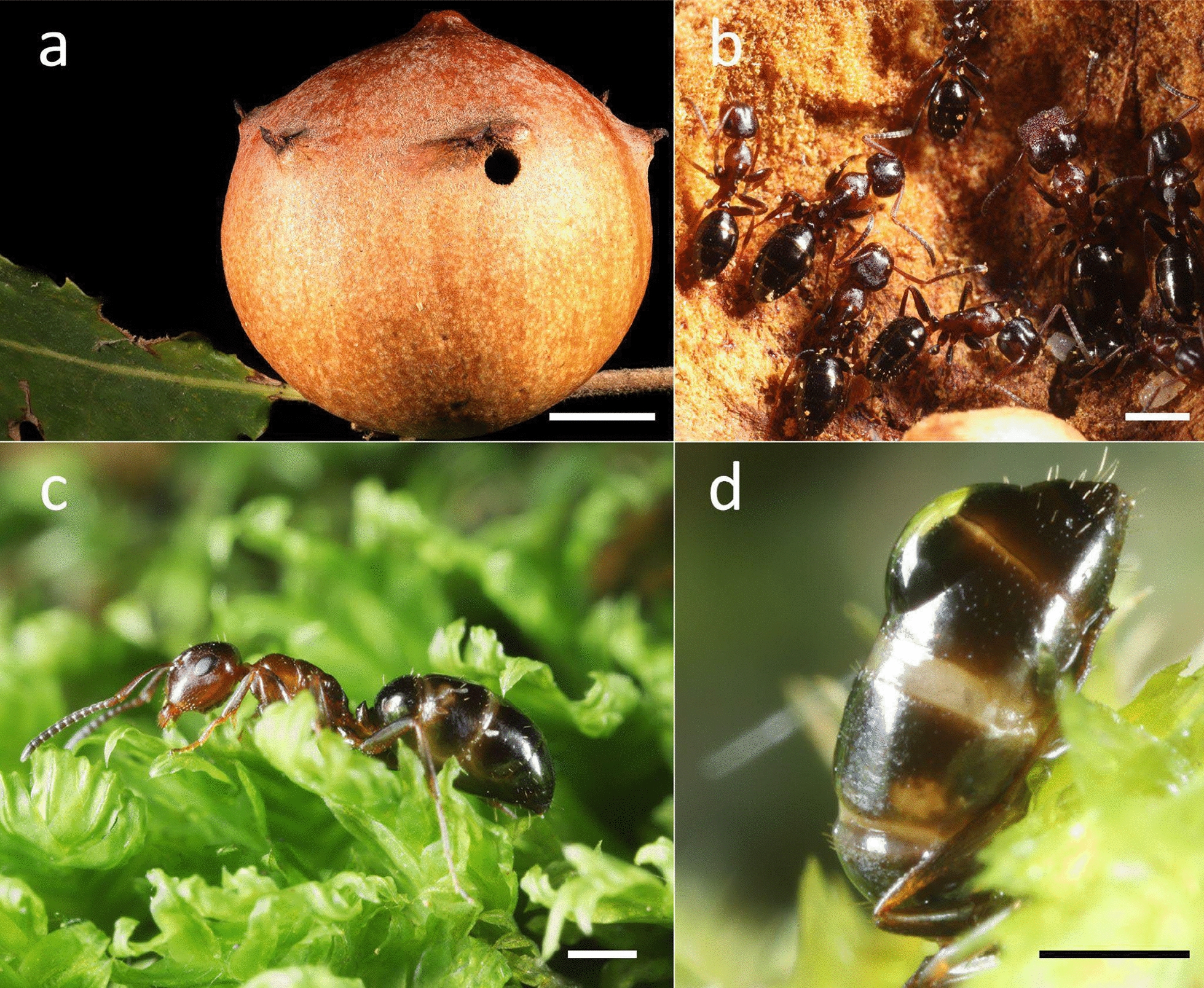
**a** Oak gall induced by the Cypinidae *Andricus quercustozae* **b** A colony of the ant *Colobopsis truncata* inside a gall induced by *A. quercustozae*  **c** Minor worker of *Co. truncata*. **d** Abdomen of a minor worker of *Co. truncata* with a drop of water. Scale bars: a, b = 1 cm; c, d = 0.5 mm

Oak galls, particularly those induced by cynipid wasps (Insecta; Hymenoptera; Cynipidae) of *Andricus kollari* (Hartig, 1843) and *Andricus quercustozae* (Bosc, 1792) (Fig. 1a), are often colonized by wood-nesting ants in temperate regions, either to establish colonies or as colony outstations. These structures represent suitable nesting sites allowing for an abundant and stable presence of ants on the host plant. Moreover, galls represent an ideal experimental setting to provide information on phoresies mediated by ants, since they are a confined type of ant nest and are easy to being experimentally isolated in order to verify the secondary occurrence of other animals. A total of 12 ant species, belonging to five different genera, were recorded nesting inside galls across Europe. The most frequent was *Crematogaster scutellaris* (Olivier, 1792), followed by *Colobopsis truncata* (Spinola, 1808), and several species belonging to the genus *Temnothorax* [12, 13]. Galls initially represent a cost for the plant. However, the arrival of secondary colonizers, especially ants, can compensate by offering significant advantages. Indeed, ants can be a long, stable presence on plants, thereby contributing to different services such as defense against phytophagous insects and pathogens [13–16].

Tardigrades are cosmopolitan microscopic animals occurring all over the world in a variety of habitats, such as permanent and ephemeral marine and freshwater bodies, but also terrestrial habitats such as moss-cushions, lichens, and leaf-litters [17]. Despite being aquatic animals, they can live in terrestrial environments thanks to their ability to enter anhydrobiosis. In the anhydrobiotic state, tardigrades are extremely dehydrated and do not show any metabolic rate but retain the ability to resume their metabolism after rehydration [18–20]. When tardigrades are dry, they compact their bodies in the so-called tun shape to better withstand various environmental stresses [21–24]. Tardigrades do not disperse efficiently over long distances relying on their own motility, and their distribution can be aided through passive dispersal mediated by waterbody streaming [25] and powerful winds [26–30]. For instance, specimens of *Echiniscus* genus have been observed in Greenland, often associated with rainfall caused by the hot, dry Foehn storms [31]. Antarctic tardigrades have been experimentally tested to wind dispersal with positive evidence [27], and in Central Europe tardigrades have shown to be transported by wind and their dispersal subject to strong seasonal dynamics [28].

The phoresy of tardigrades has been described on different species of vertebrates [32–35]. Tardigrades were observed on algae and biofilm material on the dorsal carapace of turtles [36] while some migrating birds interact with tardigrade habitats during nest-building and foraging activities [33], carrying active and desiccated tardigrades and eggs in their feathers [32]. Tardigrades have also been found in birds' feces [34] and in the fresh mud left behind by wild boars on the trees they had rubbed themselves against [37]. Moreover, in the ice-free areas of Antarctica, freshwater tardigrades are transported on the footwear of tourists and researchers, thereby revealing even a human-mediated dispersal [35]. Conversely, the phoresy of tardigrades by invertebrates is only demonstrated in species with reduced mobility such as gastropods: tardigrades were retrieved in the mucus [38], the digestive tract [39, 40], and the feces of snails [41]. Tardigrades can survive passing through the gastrointestinal tract of a terrestrial gastropod, and later reproduce [42]. According to Greven [43], Heinis [44] occasionally found tardigrades or their eggs on gastropods, myriapods and beetles. Although wind and water are generally assumed to be the dominant dispersal mechanisms for tardigrades, further pathways of endo- or ectozoochory remain to be investigated.

In our research we studied interactions between ants and tardigrades. We asked whether the ants have an active role in the dispersion of tardigrades. We carried out the following steps: i) a field survey to investigate the potential presence of tardigrades inside *Quercus* spp. galls induced by the cynipid wasp *A. quercustozae* that had been colonized by ants; ii) a field investigation on the transport of tardigrades on the surface or within the galls by ants; iii) a laboratory experiment to analyze the potential mechanisms of phoresy behavior of ants dispersing tardigrades.

## Materials and methods

The study area was in a mixed oak forest (*Quercus* spp.) in the Lunigiana area (Northern Tuscany, Italy) near the village of Fornoli (1 km^2^ in the surroundings of 44°15′24" N, 9°58′08" E), where galls induced by *A. quercustozae* were present.

### Field survey of tardigrades inside the galls

A field survey was performed to verify hypothetical and potential tardigrade-ant associations inside galls of *Quercus* spp. colonized by ants, and to select promising ant species for subsequent experiments. This survey was conducted from March to May 2024 and comprised the selection and collection of 129 gall samples induced by *A. quercustozae* that bore the typical oak gall wasp’s hole (i.e. abandoned by the gall-inducer) using methods described in [13]. The galls collected from 40 trees (in an area of three hectares) were sectioned to evaluate the species and composition of the ant colonies [13]. The external and internal portions of each gall were scratched and placed in distilled water for 30 min to evaluate the presence of tardigrades. The water of each gall portion was then sieved (meshes of sieves: 500 μm and 38 μm) to extract potential tardigrades and their eggs.

The collected ants were frozen, air dried, and observed under a stereomicroscope (ZEISS Stemi 508, 5–200X magnification range with Axiocam Erc 5s) to take morphometric measurements and to identify the species according to the identification keys [45, 46]. Each ant was checked for tardigrade presence on its body and imaged under a stereomicroscope (Leica MZ125, 8–100X magnification range with AmScope MU1803).

All tardigrade specimens were mounted on permanent slides in Hoyer’s mounting medium for observation with a light microscope [47]. Tardigrades taxa identification was performed with a light microscope (Leica DM RB with AmScope MU1803 or digital Keyence VHX-7000N) according to the taxonomic literature [48–51].

### Effects of ant colonization on the presence of tardigrades

To evaluate the transport of tardigrades by ants within their gall nests an experiment with oak galls was set up. Two oak trees (T1: 44°15′40.2" N, 9°58′18.3" E; T2: 44°15′34.2" N, 9°58′10.0" E) with galls were chosen in the study area. A set of 43 oak galls at 3–5 m from the bases of the trunk of T1 and T2 were selected among those not having the typical gall wasp’s hole, meaning that the cynipid (*A. quercustozae*) was still inside [13]. In March 2022, these galls were isolated using a safety net preventing colonization by ants or other arthropods once the wasp emerged [13].

One year later (March 2023), only 22 galls, of those isolated on the two trees (T1 and T2), bore the typical oak gall wasp’s hole, showing that they had already been abandoned by the gall-inducer. The safety nets were removed from these galls. Ten of these galls were used as control group: these galls were maintained isolated with adhesive strips at the base of the branch to prevent colonization by ants. After one additional year (March 2024) during which the 12 experimental galls were exposed to ant colonization, the galls were collected. At the end of the experiment, four control galls were discarded as they were broken. The analyses of the galls to observe ant colonies and tardigrade presence, the preparation and identification of ants, and the identification of tardigrades were carried out as mentioned in the paragraph named “*Field survey of tardigrades inside the galls*”. The tardigrades retrieved on the ant bodies were prepared together with the relative host ants for Scanning Electron Microscope (SEM; protocol B3 by Camarda et al., 2023 [52]).

Retrieved data about the possible presence of tardigrades within the galls colonized by ants were statistically analyzed via Chi-square test with Yate’s correction. Moreover, to evaluate whether the presence of tardigrades was significantly associated with one of the analyzed ant species, the Mann Whitney U test was performed.

### Ant foraging distances from the nest and presence of tardigrades in the foraging areas

To evaluate the possible collection and transport of tardigrades by ants during their foraging travels an experiment with ants was performed. Before the collection of the galls selected for the experiment performed in the paragraph “*Effects of ant colonization on the presence of tardigrades*”, several ants had been marked as soon as they had emerged from the experimental galls to quantify the foraging distance covered [from each gall: *Co. truncata*, 15 minor workers (Fig. 1b-c); *Cr. scutellaris*, 15 workers]. These distances were measured through a laser distance meter to assess the potential transport of tardigrades. The distance for the workers that forage in the canopy was not quantified due to the impossibility to track their path among the branches with the method used. One moss sample (~ 25 cm^2^) per tree trunk (T1 and T2) was collected on the trail followed during ant foraging and analyzed to identify the tardigrade species.

### Experimental validation of the tardigrade-ant phoresy

**Fig. 2:**
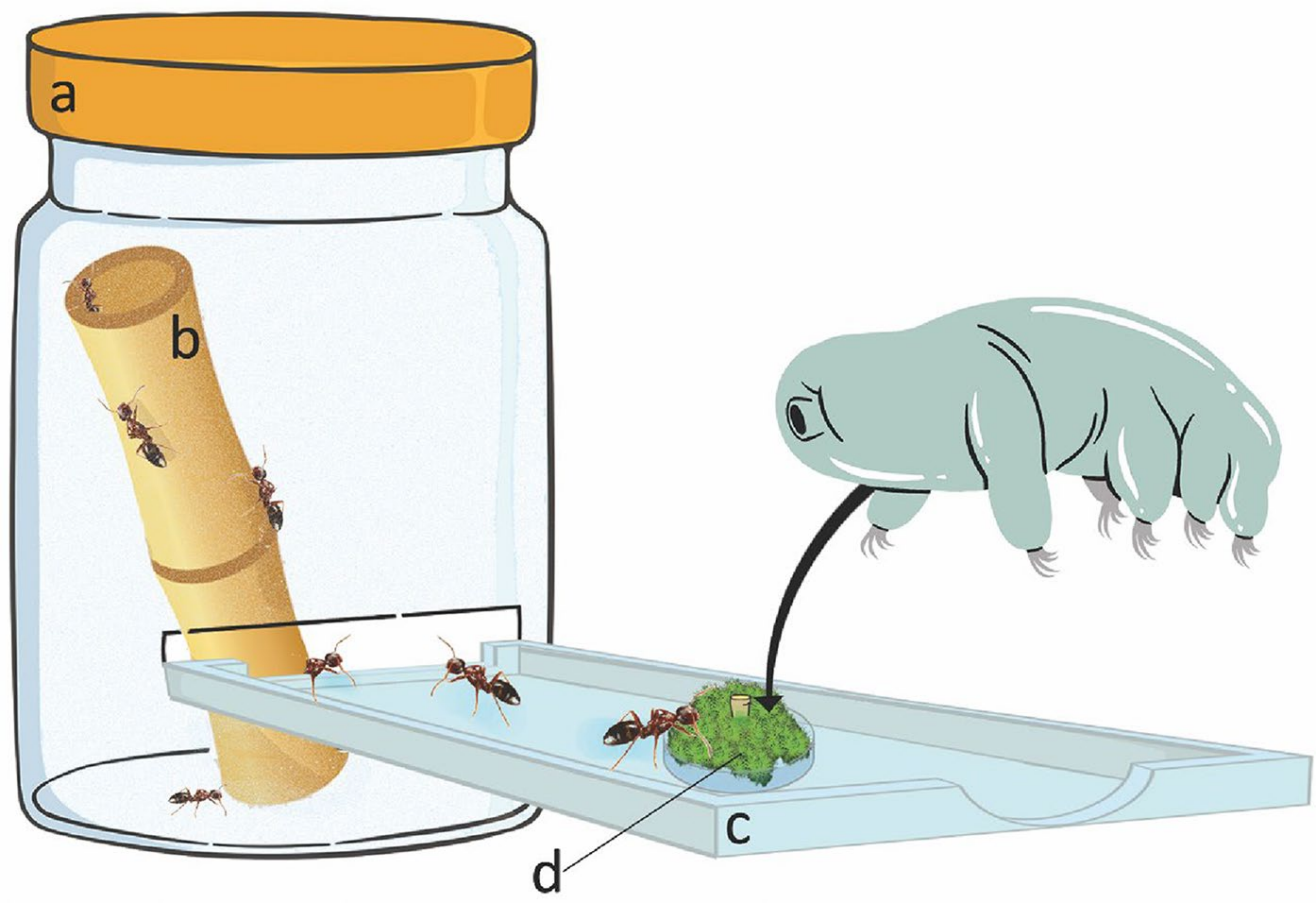
Schematic representation of the experimental apparatus with workers of *Colobopsis truncata,* composed of a nest area **a**, a dry bamboo stick **b**, an experimental arena **c**, and a patrolling facility **d**.

A laboratory experiment was carried out to analyze the potential mechanisms of phoresy behavior of the ants dispersing tardigrades. In the study area, some galls colonized by the ant *Co. truncata* were collected to obtain three entire ant colonies (*i.e.* composed of queen, workers, and brood). Five days before the experiments, these colonies were located in artificial nests [diameter (ø): 6 cm; height (H): 9 cm; Fig. 2] with natural nest fragments at standardized rearing conditions (temperature: 25 ± 1 °C; relative humidity: 50 ± 5%; photoperiod: 16/8 h light/dark). Colonies were fed with a honey-water solution (70% organic honey solution) and with *Tenebrio molitor* Linnaeus, 1,758 (Insecta; Coleoptera) larvae ad libitum. Three experimental apparatuses were set up (Fig. 2), each representing a replicate of the experiment (one for each colony) and composed of a nest area (Fig. 2a) with a dry bamboo stick (Fig. 2b) and an experimental arena (Fig. 2c) contained a feeder surrounded by a moss fragment inhabited by meiofaunal animals (henceforth referred to as the 'patrolling facility', Fig. 2d). A plastic box (ø = 6 cm, H = 9 cm) was used as the nest area and was connected with the experimental arena [length (L): 7 cm; width (W): 5 cm; H: 3 cm]. The stick of bamboo (ø = 4 cm, L = 6 cm), which had previously been sterilized with autoclave and washed with sterilized water to eliminate any potential presence of organisms, was placed inside the nest area, as an experimental nest for ants. Furthermore, the plastic parts of the device were rinsed with sterile water. We placed the patrolling facility in the experimental arena on the opposite side of the nest area. This was composed of a small plastic petri dish (ø = 15 mm; H = 7 mm) stuffed with a fragment of wet moss cushion (corresponding to 0.5 gr dry weight) collected at Modena (Italy).The bottom portion of a 0.5 ml plastic tube was inserted in the middle of the moss fragment as a feeder, this was filled with honey-water solution to facilitate the patrolling of ants on the moss. The moss fragment, which hosted about 150 animals (moss tardigrades density of 300 animals/gr; E. Massa personal communication), was artificially overpopulated by adding more than 150 tardigrades of different taxa to those already inhabiting the fragment. The density of tardigrades within a moss can vary a lot; it can be common to find around 400–500 animals/gr (e.g. [53–55]) up to 4600 animals/gr [54]. Therefore, the addition of these tardigrades did not lead to exceed the potential number of animals that ants can encounter in nature. This moss was inhabited by rotifers, nematodes, and five tardigrade species: *Macrobiotus* cf. *sapiens*, *Mesobiotus* sp., *Minibiotus* sp., *Ramazzottius* sp., and *Echiniscus* sp. The additional tardigrades were collected as described above from the remaining moss sample from which the fragment was separated to set the arena.

At the onset of the experiment, ants were allowed to exit the nest area to freely move around the experimental apparatus for 24 h. The experimental apparatuses were maintained in a climatic chamber at 26 °C with a photoperiod of 16/8 h light/dark for the whole duration of the experiment. At the end of the experiment, the apparatuses were frozen (− 20 °C for 24 h). Then all components of the apparatuses and each single ant were checked in order to evaluate: i. the presence of tardigrades on the body of the ants; ii. the presence of rotifer, nematodes, and tardigrades inside the bamboo sticks (Fig. 2b); iii. the presence of rotifers, nematodes, and tardigrades in the nest area (Fig. 2a) or in the experimental arena (Fig. 2c).

All the retrieved tardigrades were mounted on permanent slides following the methods described in the paragraph “*Field survey of tardigrades inside the galls”*. The same procedure was applied for the tardigrade found on the abdomen of one ant: the tardigrade was first rehydrated along with the ant in a Boveri capsule using distilled water and then both animals were mounted on the same slide. All slides were subsequently observed under a light microscope for species identification.

## Results

### Field survey of tardigrades inside the galls

The gall content analysis showed that 83 galls were colonized by ants, 10 were colonized by spiders, and 36 were empty. Four galls were found broken and were therefore excluded from the analysis. Four ant species were identified: *Cr. scutellaris* was the most abundant species, colonizing 40% of galls (50 galls); *Co. truncata* (17 galls); *Temnothorax italicus* (Consani, 1952) (9 galls); *Camponotus fallax* (Nylander, 1856) (3 galls).

The analysis of the material extracted from the internal portion of the galls showed the presence of tardigrades in 5 galls colonized by *Co. truncata* (Table 1). Tardigrades were not found in the material sieved from the external portion of any gall.

**Table 1:** Content of the galls collected during the field survey

Ant species colonizing galls	Ant colony composition	Tardigrade taxa (number of specimens)
*Co. truncata*	Workers + brood	*Macrobiotus* gr. *hufelandi* (1)
		*Mesocrista* sp. (1)
*Co. truncata*	Queen + workers + brood	*Macrobiotus* gr. *hufelandi* (1)
*Co. truncata*	Queen + workers + brood	Hypsibidae sp. (1)
*Co. truncata*	Queen + workers + brood	*Mesocrista* sp. (1)
*Co. truncata*	Queen + workers + brood	*Mesocrista* sp. (1)
		*Macrobiotus* gr. *hufelandi* (1)
*T. italicus*	Queen + workers + brood	*Macrobiotus* sp.* (1)

^*^Tardigrade retrieved in the lichen fragment

The analysis also revealed the presence of moss and lichen fragments inside several galls colonized by *Co. truncata* and *T. italicus*. Tardigrade specimens of *Macrobiotus* sp. were found in a fragment of a lichen inside the gall nest of *T. italicus.* The lichen fragments could be part of the construction materials functional to reduce the entrance hole of the galls. Indeed, as described by Giannetti et al. [56], *T. italicus* mixes dead insect fragments (sometimes including defensive hastisetae of dermestid larvae) to reduce the size of the entrance hole of galls.

### Effects of ant colonization on the presence of tardigrades

The results of the gall content analysis showed that 11 out of the 12 experimental galls were colonized by complete colonies of ants: 1 gall by *Cr. scutellaris*, 6 by *Co. truncata*, 2 by *Ca. fallax*, and 2 by *Dolichoderus quadripunctatus* (Linnaeus, 1771). The colonized galls with ants were compared with the six control galls from which ants were excluded. The examination of the galls colonized by *Co. truncata* (Table 2) revealed the presence of six tardigrade taxa in five out of the six galls*.* Furthermore, when analyzing the internal portion of the galls hosting *Co. truncata*, we recorded the presence of eight live tardigrades, together with moss and lichen fragments, within the ant nests. The statistical analysis showed a significant difference between the ant-colonized galls and the control galls in the tardigrade presence (p < 0.01; Chi^2^ = 10.549; n = 18). A significant association of tardigrade presence in galls colonized by *Co. truncata* was also found (p < 0.05; W = 27; n = 11).

**Table 2:** Content of the colonized galls and tardigrades inside moss samples collected during the continuous monitoring of the ants on the two oak trees

Ant species colonizing galls	Tree	Ant colony composition	Tardigrade taxa in galls (number of specimens)	Tardigrade taxa in moss on tree (number of specimens)
*Cr. scutellaris*	T1	Queen + workers + brood	*Macrobiotus* sp. (1) *	*Echiniscus* sp. (3) *Hypsibius* sp. (1) *Mesobiotus* sp. (4) *Paramacrobiotus* sp. (1) *Macrobiotus* sp. (26)
*Co. truncata*	T1	Queen + workers	Hypsibiidae (2) *	
			*Minibiotus* sp. (1)	
*Co. truncata*	T1	Queen + workers	*Macrobiotus* sp. (5) *Minibiotus* sp. (1) *Milnesium* sp. (1)	
*Co. truncata*	T2	Queen + workers + brood	*Paramacrobiotus* sp. (1 egg)	*Ramazzottius* sp. (2) *Paramacrobiotus* sp. (4)
			*Paramacrobiotus* sp. (1)	
*Co. truncata*	T2	Queen + workers	*Paramacrobiotus* sp. (2)	
*Co. truncata*	T2	Queen + workers + brood	*Macrobiotus* sp. (3)	
			*Mesobiotus* sp. (2)	
			*Milnesium* sp. (3)	

^*^Tardigrades retrieved on ant abdomens

**Fig. 3:**
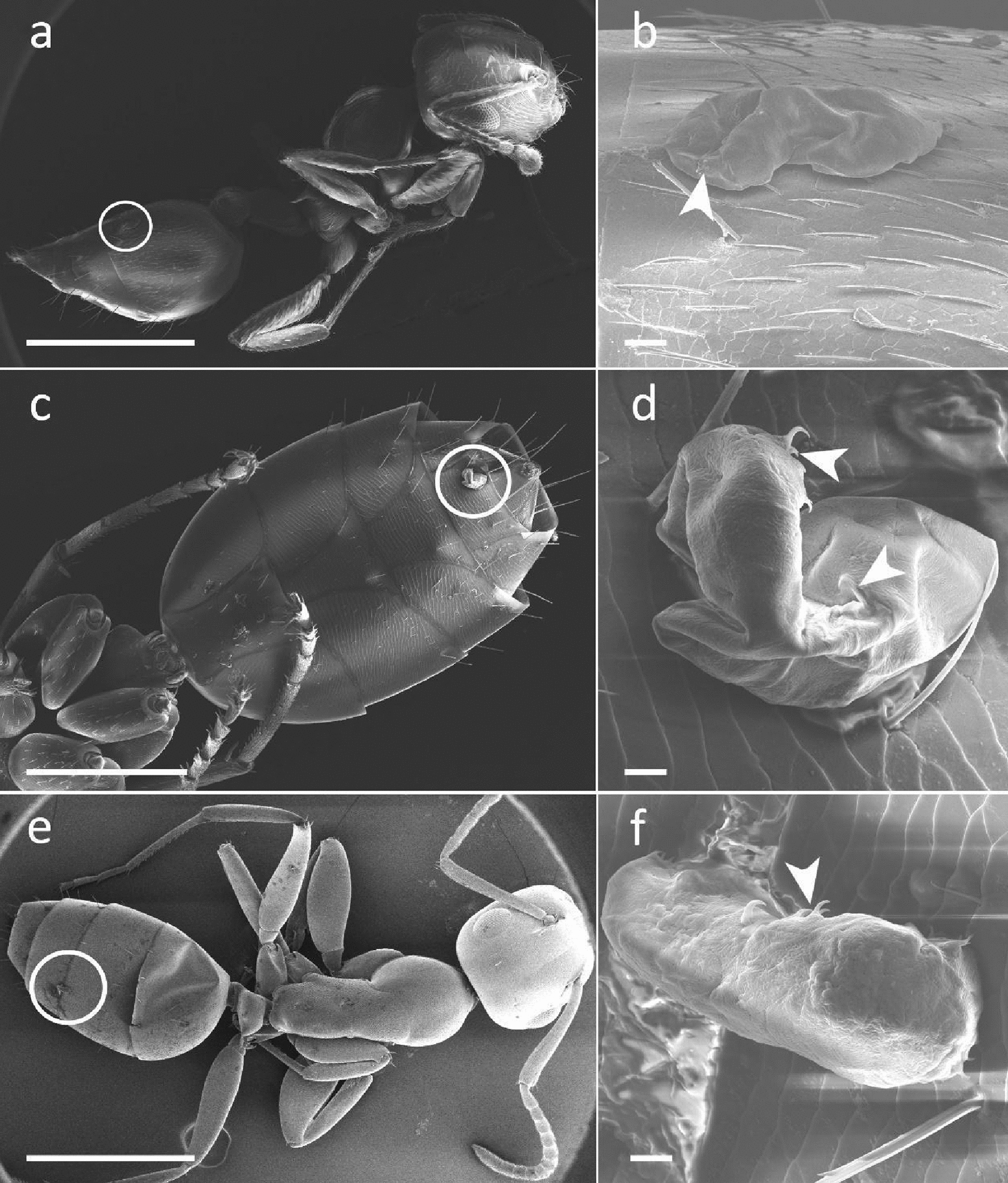
Tardigrades on the ant abdomens (SEM). Left-hand images **a**, **c**, **e**: workers of *Crematogaster scutellari*s **a** and *Colobopsis truncata* **c**, **e**. Right-hand images **b**, **d**, **f**: enlargements of the tardigrades within the circles in the corresponding left-hand images **a**, **c**, **e**. Arrowheads: tardigrade claws. Scale bars: a, c, e = 1 mm; b, d, f = 10 µm

Moreover, we recorded the presence of two tardigrades (Fig. 3d, f) belonging to the family Hypsibiidae, each on the abdomen of two *Co. truncata* workers, one on the ventral and one on the dorsal side (Fig. 3c, e). The presence of tardigrades was instead not observed on the external surface of any gall. Only in a gall of *Cr. scutellaris*, a specimen of *Macrobiotus* sp. (Fig. 3b) was found on the ventral side of the abdomen of a worker (Fig. 3a), even though tardigrades were not retrieved in the internal or external portion of the galls. No moss/lichen fragments or tardigrades were found in the control galls, or in the galls with only the queen of *Cr. scutellaris*, *Ca. fallax*, and *D. quadripunctatus*.

### Ant foraging distances from the nest and presence of tardigrades in the foraging areas

During the continuous monitoring of ants, the abdomen of *Co. truncata* was observed to be lowered and therefore in contact with the substrate (moss and/or soil), and the presence of drops of water (Fig. 1d) both on the upper and the lower side of *Co. truncata* abdomens was recorded.

The foraging distance measurements showed an average of 10.3 m (SD = 4.5) reached by the workers of *Co. truncata* with a maximum distance of 21.0 m. Instead, a foraging mean distance of 10.3 m (SD = 3.5) was recorded for *Cr. scutellaris*, with a maximum distance of 19.0 m. The mosses sampled on the ant trails on the trunk of the two trees (T1 and T2; on which the colonized galls were collected) were inhabited by six tardigrades taxa reported in Table 2.

### Experimental validation of the tardigrade-ant phoresy

**Fig. 4:**
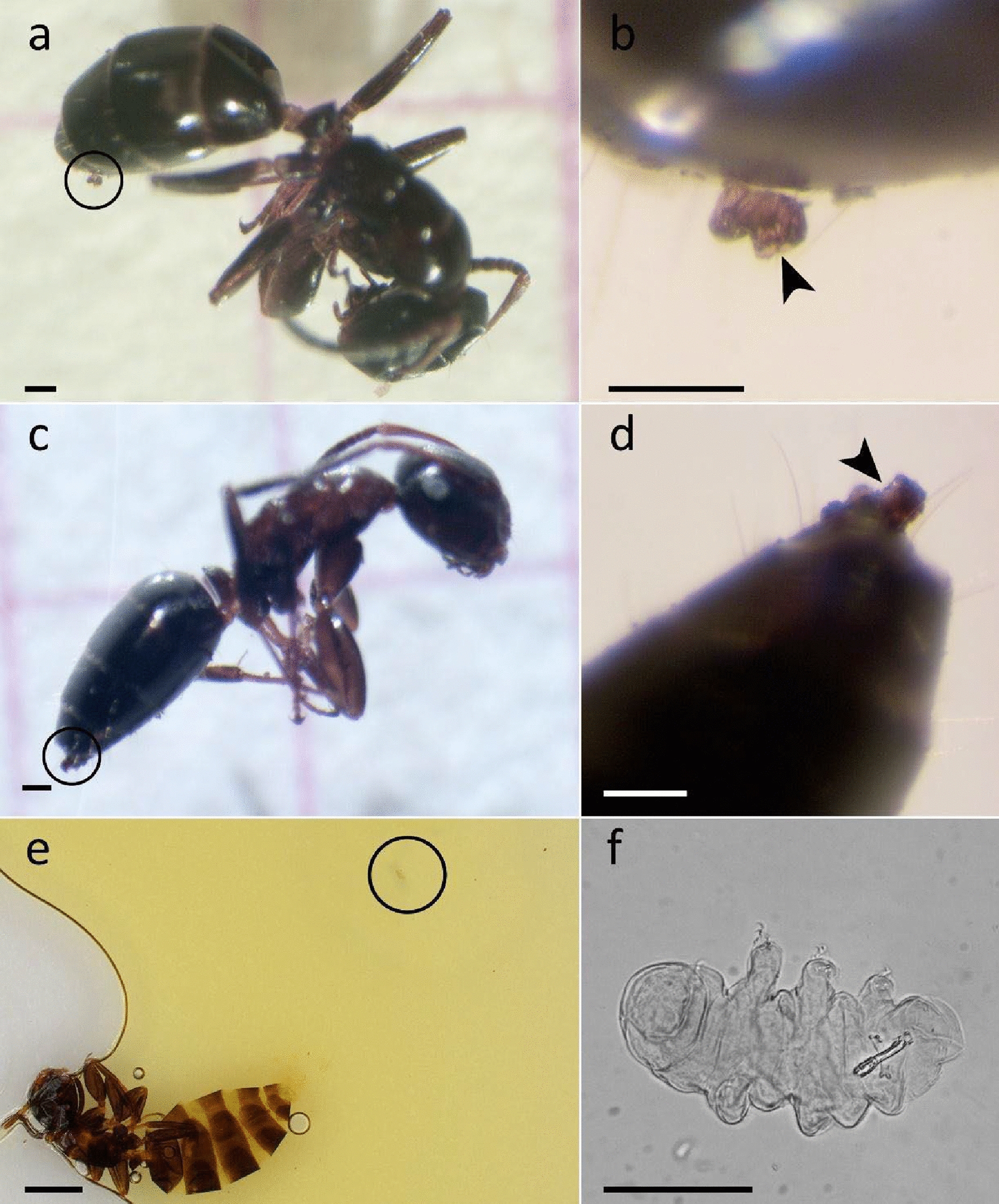
*Colobopsis truncata* minor workers **a**, **c** with tardigrades on their abdomen **b**, **d**. *Co. truncata* minor workers **e** along with a tardigrade **f** retrieved on its abdomen and mounted together on slide after rehydration. Right-hand images **b**, **d**, **f**: enlargements of the tardigrades within the circles in the corresponding left-hand images **a**, **c**, **e**. a-d: stereomicroscope, e: digital light microscope, f: light microscope. Scale bars: a, c, b, d, f = 100 µm; e = 1 mm

The presence of tardigrades, rotifers, and nematodes on the experimental apparatuses was checked along with tardigrades on the surfaces of ant bodies. The presence of tardigrades was recorded among all the analyzed items (Table 3). Both living and dead tardigrades were found in the bamboo stick, along with dead nematodes and living and dead rotifers. The nest area and the experimental arenas hosted both live and dead tardigrades and rotifers. The observation of the ant body surfaces revealed the presence of tardigrades in two out of the three experimental settings. Three tardigrades (Fig. 4b, d, f) were retrieved on the abdomen of three worker ants (Table 3; Fig. 4a, c, e). Moss fragments were displaced by ants in the different parts of the three experimental apparatuses.

**Table 3:** Tardigrades, nematodes, and rotifers retrieved in the laboratory experiment with *Co. truncata*

Experimental replicates	Analyzed items	Number of tardigrades	Other taxa
1	Ant bodies	2	
	Nest area + Experimental arena	2 (dead)	2 rotifers (1 alive, 1 dead)
	Bamboo stick	2 (1 alive, 1 dead)	1 rotifer (dead)
2	Ant bodies	1	
	Nest area + Experimental arena	2 (1 alive, 1 dead)	
	Bamboo stick	4 (dead)	1 nematode (dead)
3	Ant bodies		
	Nest area + Experimental arena	2 (1 alive, 1 dead)	several rotifers (dead)
	Bamboo stick	2 (1 alive, 1 dead)	1 nematode (dead)

## Discussion

The analysis has revealed for the first time the role of ants as potential dispersal hosts for different taxa of tardigrades (and other terrestrial meiofauna) and their transport inside the galls. The study conducted on four arboreal ant species that colonize the galls has shown the presence of tardigrades inside the nests of three of them. *Co. truncata* showed the greatest transport capacity, exhibiting the presence of tardigrades on its body too. The frequent presence of water drops on the abdomen of *Co. truncata,* due to a lowered posture during walking, and the transport of materials such as moss and lichen fragments can explain the transportation of tardigrades. The reduced grooming behavior, which seems to be less frequent in *Co. truncata* compared to *Cr. scutellaris* and *T. italicus*, and the speed of workers (2.4 cm/sec), guarantee that the substrate fragment picked up by the ant, if wet, remains hydrated during the transport toward the nest inside the gall. The foraging distance walked by ant allows tardigrades, nematodes, and rotifers (identified within the galls) to be potentially passively dispersed by ants for relatively long distance (about 10 m) each time an ant leaves the nest. Moreover, animals can be dispersed over a longer distance if they are in a desiccated state. Indeed, they could be in an anhydrobiotic state within a dry substrate fragment transported by the ants, or they could properly desiccate on the body of an ant, if the process occurs slowly and under high humidity environmental conditions [18].

The low numbers of tardigrades observed on ant abdomens suggest that this phoresy may be a rare occurrence rather than a common phenomenon. Actually, our data are comparable to those previously obtained for the dispersal of tardigrades by wind. Ptatscheck et al. [28] reported a maximum dispersal rate (individuals m^-2^) of 64 tardigrades over a four-week experiment, corresponding to a mean of 16 animals per week. Likewise, Janiec [57] found a total of 182 tardigrades across 11 sites during a six-week experiment, corresponding to a mean of 2.8 tardigrades per site per week.

Our data suggests that this mechanism can assure a faster dispersal compared to other zoochore dispersals: e.g. gastropods transporting tardigrades cover 10 m in 48 h [42]. Compared to other transport mechanisms ants can ensure a stable presence in the area and consistent patrolling activity on both the ground and plants.

The foraging distance walked by the ants and the high number of individuals in the colonies, potentially covering a conspicuous area, could also explain the diversity of the tardigrades retrieved within the galls. Indeed, there were differences in the taxa of tardigrades found in the galls and those extracted from the mosses collected on the plants where the galls were sampled (Table 2). For instance, the xerophile and eurytopic tardigrades of the genera *Ramazzottius*, *Milnesium*, and *Echiniscus* are often retrieved on substrates undergoing frequent water fluctuation [57] and could therefore be “bycaught” on mosses of the trunk, while retrieved specimens of *Mesocrista* are more likely found in hydrophilic habitats [58], such as the leaf litter where ants patrol. The other tardigrade taxa (as *Minibiotus, Macrobiotus, Paramacrobiotus, Mesobiotus*; Macrobiotidae) can be found in both types of environments [58].

Furthermore, the galls represent a new site of colonization by tardigrades. They are suitable ant nest sites allowing an abundant and stable presence of ants on the host plant [13, 15, 56]. The humidity fluctuations within the galls due to weather conditions provide a suitable environment for active tardigrades and could allow slow dehydration, enabling the mechanism of anhydrobiosis. Moreover, hitchhiking tardigrades could be released on other substrates on the ant’s route, thereby rising habitats potentially colonized by them and increasing their genetic connectivity among different substrates. Although a low frequency of transport on worker ants was observed in the wild, the presence of tardigrades within the galls highlights its potential efficiency. However, the constant presence and activity of ants in the area surrounding the gall nests may increase the likelihood of transport, as demonstrated in laboratory experiments. The presence of dead tardigrades found in the experimental apparatuses may be attributed to the quick-freezing methodology used, which, while making it possible to demonstrate the transport of the tardigrades, could have affected the viability of tardigrade species.

Overall, this work on ant arboreal species opens new perspectives for the study of the role of ants as hosts in the transport and dispersal of tardigrades and other micrometazoans. Due to the distribution of tardigrades and ants across different environmental matrices the chances of these two animals meeting each other are enormous, so further investigations may clarify for instance the role of soil-dwelling ant species in the transport of the tardigrade-sized or smaller organisms. Thanks to their huge evolutive and ecological radiation, ants count about 14,000 species with billions of individuals, representing 30–45% of the entire insect biomass of the world [59], which move around on different kinds of substrates, and potentially connect many different habitats three-dimensionally. This myrmecophoretic dispersion could have huge importance in the spreading of tardigrades and other meiofaunal organisms in terrestrial environments and our finding might represent just the’tip of the iceberg’ of an unexplored passive dispersal modality for terrestrial micrometazoans.

## Data Availability

All data generated for this study are displayed in the main text.
